# Post-traumatic Orbital Abscess in an Adult With No Evidence of Orbital Fracture, Paranasal Sinusitis, or Foreign Body Migration

**DOI:** 10.7759/cureus.13376

**Published:** 2021-02-16

**Authors:** Yasuhiro Takahashi, Satoshi Kakutani, Aric Vaidya, Hirohiko Kakizaki

**Affiliations:** 1 Oculoplastic, Orbital and Lacrimal Surgery, Aichi Medical University Hospital, Aichi, JPN; 2 Plastic Surgery, Uji Takeda Hospital, Kyoto, JPN; 3 Ophthalmology, Rapti Eye Hospital, Dang, NPL

**Keywords:** orbital abscess, orbital fracture, foreign body, paranasal sinusitis, emergent drainage, intravenous antibiotic

## Abstract

A 35-year-old man was hit against his left eye by his child’s foot. Two days following trauma, the patient noticed diplopia in the upward and right gazes. On the first examination seven days after trauma, computed tomographic (CT) images revealed a small mass in the inferolateral orbit near the inferior orbital fissure. There was no radiological evidence of orbital fracture, paranasal sinusitis, or foreign body. Immediately after the first examination, the patient had a history of fever, retrobulbar pain, and nausea. These symptoms gradually worsened, and the patient visited the emergency department of our hospital at 13 days following trauma. CT images showed enlargement in the size of the mass. The diagnosis of the orbital abscess was made, and emergent drainage of the abscess was performed, followed by administration of intravenous antibiotics. On the fifth postoperative day, cultures of the abscess specimen showed growth of *Fusobacterium nucleatum* (4+), *Parvimonas micra* (4+), and *Prevotella intermedia* (4+). The patient’s condition improved significantly and at the 1.5-month follow-up, the patient did not have any symptoms related to the orbital abscess.

## Introduction

An orbital abscess is a rare entity potentially causing blindness with serious life-threatening complications, which necessitate urgent attention [1]. The most common cause of orbital abscess in adults is periocular trauma, although it occurs less frequently in adults [2-5]. The orbital fracture can compromise the blood supply to the orbital fat, which allows anaerobic orbital infection [6,7]. In patients with preexisting paranasal sinusitis, the infection can directly extend into the orbit through the fracture site [3,8]. Similarly, foreign body migration can also be a source of the orbital abscess [3].

Here, we report a rare case of orbital abscess after blunt periocular trauma with no evidence of orbital fracture, paranasal sinusitis, or foreign body migration.

## Case presentation

This case report adheres to the tenets of the 1964 Declaration of Helsinki. Written informed consent for the publication of this report and identifiable patient photos were obtained from the patient.

A 35-year-old man had a history of trauma to his left eye with his child’s foot. Two days after the accident, the patient noticed diplopia in the upward and right gazes. He had no past history of any immunocompromised disease, ocular or periocular surgery, or paranasal sinusitis. There were no symptoms regarding dental infection, mid-ear otitis, or upper respiratory tract infection.

**Figure 1:**
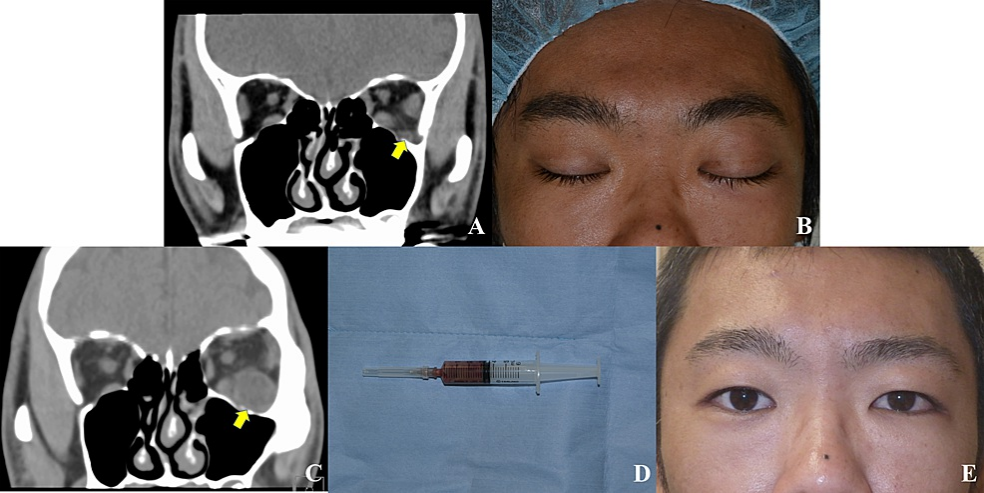
Case presentation. (A) An axial computed tomographic image taken on the first examination showing an orbital mass (arrow) near the inferior orbital fissure. (B) A face photo taken before emergent surgery. The eyelid is swollen on the left side. (C) An axial computed tomographic image taken before emergent surgery showing an enlargement of the orbital mass (arrow). (D) Aspirated orbital abscess. (E) A face photo taken at 1.5 months follow-up showing no swelling in the eyelid and cheek.

On the first examination seven days after trauma, his best-corrected visual acuity was 1.2 in both eyes, and intraocular pressure was 13 mmHg in the right eye and 18 mmHg in the left eye. Hertel exophthalmometric value was 12 mm in the right eye and 12.5 mm in the left eye (base, 104 mm). The result of the Hess chart showed restriction of supraduction and adduction in the left eye, and the result of the field of binocular single vision demonstrated diplopia in the primary and upward gazes. Computed tomographic (CT) images revealed a small mass in the inferolateral orbit near the inferior orbital fissure (Figure 1A). The lateral and inferior rectus muscles were enlarged. There was no radiological evidence of orbital fracture, paranasal sinusitis, or foreign body.

Post-traumatic orbital inflammation and hematoma were suspected. At the patient’s request, 50 mg/day of oral prednisolone was prescribed for the quick recovery of diplopia.

Immediately after the first examination, the patient had a history of fever, retrobulbar pain, and nausea. These symptoms gradually worsened because of which the patient visited the emergency department of our hospital at 13 days following trauma. At that time, he took non-steroidal anti-inflammatory drugs to reduce pain.

On examination, the light reflex was prompt, and the left intraocular pressure was 49.2 mmHg. Supraduction and adduction were severely restricted in the left eye. There was swelling in his left eyelid and cheek along with tenderness (Figure 1B). CT images showed that the mass was enlarged than before (Figure 1C). His body temperature was 36.9℃. Blood tests revealed an elevated white blood cell count (17,400/μL; normal range, 3,100-8,400/μL) and C-reactive protein (1.15 mg/dL; normal range, <0.3 mg/dL).

A diagnosis of the orbital abscess was made, and after hospital admission, emergent drainage of the abscess was performed under general anesthesia by two of the authors (YT and SK). After a lateral canthotomy and cantholysis, the orbital septum was incised. The abscess was drained using a syringe (Figure 1D) and was sent for a culture sensitivity test. The lesion was irrigated with saline. At this time, the left intraocular pressure reduced to 19.8 mmHg. Finally, a penrose drain was inserted. We did not close the wound to keep the left intraocular pressure reduced and prevent orbital compartment syndrome [9].

We started to administer 2 gm/day of intravenous ceftriaxone and continued to irrigate the lesion via the drain. On the third postoperative day, we changed the intravenous antibiotic to 18 gm/day of tazobactam/piperacillin, based on the advice from the infection control team. On the fifth postoperative day, cultures of the abscess specimen on routine bacterial culturing showed growth of *Fusobacterium nucleatum* (4+), *Parvimonas micra* (4+), and *Prevotella intermedia* (4+) (4+ means identification of bacteria > 30/field at the magnification of x 1,000). As all the micro-organisms were found to have high drug sensitivity for ampicillin, clindamycin, cefmetazole, amoxicillin/clavulanate, imipenem, meropenem, tazobactam/piperacillin, and moxifloxacin, we did not change the antibiotic. Dental consultation was done, but only mild periodontitis was found with no orbital extension. On the eighth postoperative day, the lateral canthus was re-fixed along with the closure of the wound. After that on postoperative 10 days, administration of intravenous antibiotic was stopped and changed to oral amoxicillin 1.5 gm/day for five days, following which the patient was discharged from the hospital.

At a 1.5-month follow-up, the patient’s best-corrected visual acuity was 1.2 in both eyes, and intraocular pressure was 15 mmHg in the left eye. Extraocular muscle motility was normal, and the patient did not notice diplopia in any direction of gaze. The eyelid and cheek swelling completely subsided (Figure 1E).

## Discussion

We report a rare case of an orbital abscess after blunt periocular trauma with no evidence of orbital fracture, paranasal sinusitis, or foreign body migration. Furthermore, the health status of our patient was not immunocompromised, which is one of the risk factors for the development of the orbital abscess in adults [2,5].

The etiology of orbital abscess in our case was uncertain. One of the possible causes was orbital hematoma after blunt trauma, which can be a source of infection [6]. Similarly, steroid treatment may also increase the risk of infection in the orbital hematoma.

In our case, the orbital abscess was located near the inferior orbital fissure, suggesting that the pathogens may enter into orbit through the fissure. On the other hand, culture tests revealed the growths of *F. nucleatum*, *P. micra*, and *P. intermedia* from the orbital abscess, which are the common pathogens of periodontal diseases. However, the patient had only mild periodontitis. Furthermore, although periodontitis usually extends first into the maxillary sinus from the dental roots and further into the orbit [3], our patient did not show maxillary sinusitis. In addition, we drained the abscess directly using a syringe and sent it for the cultural test. As the amounts of the identified pathogens were large, it was unlikely that contamination of the bacteria influenced the results of the cultural test.

Although an early diagnosis of the orbital abscess is mandatory, this is sometimes difficult to achieve. Particularly, in this case, as the CT showed no orbital fracture or paranasal sinusitis, there was a delay in the diagnosis of an orbital abscess. Furthermore, as the patient took non-steroidal anti-inflammatory drugs to reduce pain, his body temperature was not so high. Therefore, the diagnosis of orbital abscess should be comprehensively done, based on the symptoms and findings of blood tests, radiographic studies, and systemic and ophthalmic examinations.

Urgent incisional drainage of the orbital abscess is required to prevent the development of serious complications including visual loss and other lethal conditions, such as cavernous sinus thrombosis, meningitis, and cerebral abscess [1]. Particularly, in this case, a quick reduction of high left intraocular pressure was required to protect vision in the left eye [9]. Also, broad-spectrum intravenous antibiotics should be given until obtaining the results of cultural tests.

## Conclusions

We present a rare case of orbital abscess after blunt periocular trauma with no evidence of orbital fracture, paranasal sinusitis, or foreign body migration. Ophthalmologists need to be vigilant of this kind of rare case to provide proper early diagnosis and treatment and prevent the incidence of possible dreadful complications.
